# Clopidogrel Within Few Hours of Coronary Artery Bypass Grafting Does Significantly Increase the Risk of Bleeding

**DOI:** 10.4021/cr226e

**Published:** 2012-09-20

**Authors:** Emad M. Hijazi, Ghassan S Musleh

**Affiliations:** a Princess Muna AL-Hussein Cardiac Center, King Abdullah University Hospital, Faculty of Medicine, Jordan University of Science and Technology, Jordan

**Keywords:** Coronary artery bypass, Bleeding, Clopidogrel

## Abstract

**Background:**

Postoperative bleeding after coronary artery surgery is partly related to platelet dysfunction. The aim of this study was to evaluate the effects of a single loading dose of clopidogrel (300 mg) before coronary angiography on bleeding and use of blood and blood products after emergency coronary artery bypass surgery (CABG).

**Methods:**

This is a nonrandomized observational prospective study between January, 2006 till December 2009, at a university hospital, we compare the results of a cohort of 65 patients who received 300 mg clopidogrel during coronary angiography that was followed by emergency CABG (group A or study group) to a cohort of 206 patients who underwent elective coronary artery bypass surgery during the same period by the same surgeons in whom clopidogrel was stopped 7 days before surgery (Group B or control group). Emergency surgery was done because of critical coronary anatomy or because of ongoing chest pain. All patients in the two groups were kept on 100 mg of aspirin until the day of surgery. Outcome data used to compare the two groups, Chest tube drainage in first 12 hours (12 h), need for re-exploration and use of blood and blood product transfusion were prospectively collected.

**Results:**

Postoperative bleeding, reoperation rates for bleeding and use of blood products are significantly more in those who received a loading dose of clopedogril within few hours of CABG (group A) compared to those who stopped clopedogril for a week before CABG.

**Conclusions:**

Preoperative 300 mg of clopidogrel is associated with significant increase in post operative bleeding, need for surgical exploration and use of blood and blood product transfusion after CABG.

## Introduction

For secondary prevention of coronary and cerebrovascular ischemic events, many patients are now receiving long-term clopidogrel (Plavix) with or without aspirin [1-3].

Clopidogrel is a potent inhibitor of platelet aggregation that works by irreversible blockage of adenosine diphosphate (ADP) mediated platelet activation. It has been demonstrated to reduce early stent failure [4], improve outcomes in acute coronary syndromes [5], and decrease all cause cardiovascular mortality [6].

For those who need emergency coronary artery bypass surgery (CABG), it may not always be possible to stop the antiplatelets before surgery. In those cases, there is an increased risk of postoperative bleeding [7].

Reoperation rate after CABG for bleeding is approximately 2% to 3%. Half of those reoperations have no identifiable surgical cause of bleeding [8, 9] and most likely caused by coagulopathy. An increase rate in postoperative bleeding results in a higher incidence of re-exploration and blood transfusion related complications [10] as well as prolonging the patients’ length of stay [8].

Advanced age and cardiopulmonary bypass time (CPB) are factors that enhance the postoperative bleeding after CABG [8, 9].

Controversy remains about the anti-aggregation agents that affecting thrombocyte function whether it does really increase the tendency of patients to have postoperative bleeding complications [11-13].

## Methods

This is a prospective study between January, 2006 till December 2009, involved two groups of patients. Group A (65 patients) who received 300 mg of clopedogril at the time of coronary angiogram followed by emergency coronary artery bypass surgery (CABG) for critical coronary anatomy or ongoing chest pain. They were transferred to operating theatre within 2 hours of their angiography. And group B ( 206 Patients) who stopped clopedogril for a 7 days before elective coronary artery bypass surgery all patients in both groups were kept on 100 mg of aspirin till the day of surgery. All first time isolated CABGs that were done during the study period were included in the study. Those who were older than 70 years, Redo CABG, CABG plus other interventions and those who were on warfarin were excluded from the study. None of our cases in either group received low molecular weight heparin in the preoperative period. Clopidogrel was the only anti-platelet agent to be given to the patients in the emergency group. None of them received GPIIb/IIIA inhipitors, heparin or low molecular weight heparin while waiting for operation. The median time between receiving 300 mg of clopedogril and operation was 2 .15 hours (range 1.30 - 2.45 hours). All cases were done on pump, none received anti-fibrinolytic agents. Pre-operative patient characteristics and postoperative data were prospectively collected. Preoperative demographic characteristics and risk factors in both groups were prospectively collected (Table 1).

**Table 1:** Preoperative and Intraoperrative Variable

	Clopidogrel group (65 cases)	No Clopedogril group (206 cases)	P value
The average age	64.9 years (range, 39 to 83 years)	63.2 years (range, 36 to 80 years)	
Male	40	146	0.157
Female	25	60	
Age	57.3	61	0.440
Diabetes	26	100	0.228
C.R.F	1	2	0.562
P.V.D	5	15	0.548
COPD	12	29	0.389
Smoking	42	152	0.152
Heamoglubin	11.9	12.3	0.155
X Clamp time (minutes)	39	36	0.126
Bypass time (minutes)	76	83	0.190
No of grafts	2.7	3.1	0.359
Steroid use	3	5	0.294
Obesity (BMI > 25 kg/m^2^)	8	11	0.056

TIA: Transient ischemic attacks; C.R.F: Chronic renal failure; P.V.D: Peripheral vascular disease; COPD: Chronic obstructive pulmonary disease; BMI: Body mass index.

To evaluate post operative bleeding, data about chest tube drainage during the first 12 hours the need for re-exploration and use of blood and blood product transfusion were collected (Table 2).

**Table 2:** Results

	Group		Total
	Clopedogril Group	No Clopedogril Group	
Blood Transfusion			< 0.001
1 - 2	0 (0.0)	186 (90.3)	
3	20 (30.8)	20 (9.7)	
4 - 5	45 (69.2)	0 (0.0)	
FFP Transfusion			< 0.001
4 - 6	16 (24.6)	206 (100.0)	
8 - 10	49 (75.4)	0 (0.0)	
Cryo Transfusion			< 0.001
0	0 (0.0)	206 (100.0)	
6 - 8	65 (100.0)	0 (0.0)	
Platelets Transfusion			< 0.001
4.00	0 (0.0)	170 (82.5)	
6.00	25 (38.5)	36 (17.5)	
10.00	40 (61.5)	0 (0.0)	
Mediastinal drainage, mean (SD), median (mL)	1230.6 (118.5);1210.0	791.3 (117.4); 785.0	< 0.001
ITU stay (days)	3.3	3.1	0.151
Total Hospital stay (days)	7.1	7.14	0.187
Re-exploration-Number of patients	9 (13.8%)	6 (2.9%)	0.001

*Cryo. Transfusion, Blood Transfusion, FFP Transfusion, Platelets Transfusion, Platelets Transfusion = (Units)

The trigger to give blood is Haemoglubin level less than 10 g/dL.The trigger to give platelets is platelet count less than 150, we did not use sophisticated platelet function tests. The trigger to give Fresh Frozen Plasma is PT > 18. There was no clinical or laboratory evidence of preoperative bleeding disorders any patient in both groups. The study has been and approved by the ethical committee at Jordan University of Science and Technology in Jordan. The data were analyzed using the Statistical Package for Social Sciences (SPSS, version 19). Frequencies, percentages, mean, and median were used to describe the data wherever appropriate. Chi-square test was used to test the significance of the differences in the distribution of the studied variables between the two groups of patients and Mann-Whitney test to test for the significance of the differences between medians. A P-value of < 0.05 was considered statistically significant.

## Results

Postoperative data are presented in (Table 2). There was no significant difference between the haemoglubin levels between the two groups (11.9 g/dL for the clopedogril group versus 12.3 g/dL for control group), Operative data showed no statistically significant difference between the two groups regarding Bypass time and aortic cross clamp time. Regarding the number of grafts there was 2.7 grafts per case for Clopedogril group versus 3.1 grafts for the control group. The use of internal mammary artery was significantly less in the emergency group (53% (34/65) of Clopedogril group versus 86% (177/206) of the control group), and this is due to many causes as, patients hemodynamic instability during anesthesia (ECG changes, critical drop of blood pressure that require early inotrop support and to go on-pump as soon as possible), iatrogenic injury of internal mammary artery during harvesting technique, and critical left anterior descending artery (LAD) lesions with small caliber. All cases in the clopidogrel group received three or more units of packed red blood cells compared to 9.7 of the control group who needed three or more units of blood. This was statistically significant (P = 0.001). Regarding platelets' use 100% of clopedogril cases received six or more units of platelets' compared 17.5% in the control group (P = 0.001), 75% of clopedogril group received 8 or more units of fresh frozen plasma compared to 0% in the control group (P = 0.001).

Chest drainage in the first twelve hours in the clopidogrel group averaged at 1,230 ± 118.5 mL, compared to 791 ± 117.4 mL in the control group. This was also statistically significance (P = 0.001).

Eight patients (13.8%) of study group required reoperation for persistent postoperative bleeding in the study group compared to 6 patients (2**.**9%) in the control group (P = 0.001).

Two patients in group clopedogril group and four patients in control group had discrete surgical bleeding sites found at the time of reoperation (2 patients bleeding from a side branch of saphenous vein and 2 patients bleeding from a side branch of LIMA and 2 from veins in the chest wall, LIMA bed). Three patients in group A continued to bleed profusely after re-exploration and were given factor VII.

## Discussion

Antiplatelet therapy is critical in the management of coronary artery disease. For patients undergoing coronary artery bypass graft surgery (CABG), controversy remains regarding the safety of preoperative antiplatelet therapy and the optimal postoperative antiplatelet regimen to maintain graft patency and reduce ischemic events [14]. Englberger reported an increase in bleeding complications with a subsequent need for platelet and fresh frozen plasma transfusions in patients receiving clopidogrel within 3 days of surgery [15]. Yusuf et al [5] recommended that clopidogrel to be stopped 5 days before an elective CABG. Weber et al in their studies on the antiplatelet effect of clopidogrel in healthy volunteers have shown that platelet function requires seven days to recover after complete stoppage of the medication [16].

The American College of Cardiology and American Heart Association guidelines published in 2004 for CABG surgery states that “If clinical circumstances permit, clopidogrel should be withheld for five days before performance of CABG surgery [17].

Chu et al [18] in their prospective study that included 312 consecutive urgent or emergent CABG found that clopidogrel within 4 days of CABG is associated with increased blood losses and reoperation for bleeding. Furthermore, a recent study showed that as many as 5% of patients presenting for CABG may require their surgery to be done on urgent or emergency basis after exposure to clopidogrel [19]. For a patient whose CABG cannot be delayed safely, prophylactic platelet transfusion has been suggested [10]. Studies have shown that postoperative blood transfusion is an independent predictor of increased long-term mortality after cardiac surgery and, thus, have a direct impact on patient prognosis [20]. For these unwanted clopidogrel complications after coronary artery bypass surgery a reversible and direct-acting oral P2Y12-receptor antagonist (Ticagrelor),provide more consistent platelet inhibition than does clopidogrel, with more rapid onset and offset of action [21-23]. In the PLATelet inhibition and patient Outcomes (PLATO) trial, long-term reversible P2Y12 inhibition with ticagrelor was better than that with clopidogrel for the prevention of cardiovascular and total death, stent thrombosis, and myocardial infarction without an increase in the rates of major bleeding in a broad population of patients with acute coronary syndromes who were started on treatment as soon as possible after hospital admission [24]. Patients given ticagrelor had significant and clinically relevant reductions in cardiovascular and total deaths, myocardial infarction, and stent thrombosis, without an increase in risk of major bleeding [25].

### Limitation of the study

Although the data were prospectively collected, the study is not a randomized trial and therefore subjected to the selection bias. The other limitation is the fact that all the study group cases were done on emergency basis which will undoubtedly lead to an increase in the incidence of almost all untoward events. Unfortunately we could not recruit elective cases into the study group because of the clear recommendations of stopping clopedogril for at least five days in elective cases.

### Conclusion

The preoperative administration of 300 mg clopidogrel within few hours of coronary artery bypass graft surgery is associated with significant increase of post operative bleeding, surgical re-exploration and use of blood products.
